# TLR4 participates in the transmission of ethanol-induced neuroinflammation via astrocyte-derived extracellular vesicles

**DOI:** 10.1186/s12974-019-1529-x

**Published:** 2019-07-04

**Authors:** Francesc Ibáñez, Jorge Montesinos, Juan R. Ureña-Peralta, Consuelo Guerri, María Pascual

**Affiliations:** 1 Department of Molecular and Cellular Pathology of Alcohol, Centro de Investigación Príncipe Felipe, C/Eduardo Primo Yúfera 3, 46012 Valencia, Spain; 2 Department of Physiology, School of Medicine and Dentistry, University of Valencia, 46012 Valencia, Spain; 3 Department of Neurology, Columbia University Medical Center, New York, USA

**Keywords:** Extracellular vesicles, Glial cells, Ethanol, Neurons, Neuroinflammation

## Abstract

**Background:**

Current evidence indicates that extracellular vesicles (EVs) participate in intercellular signaling, and in the regulation and amplification of neuroinflammation. We have previously shown that ethanol activates glial cells through Toll-like receptor 4 (TLR4) by triggering neuroinflammation. Here, we evaluate if ethanol and the TLR4 response change the release and inflammatory content of astrocyte-derived EVs, and whether these vesicles are capable of communicating with neurons by spreading neuroinflammation.

**Methods:**

Cortical neurons and astrocytes in culture were used. EVs were isolated from the extracellular medium of the primary culture of the WT and TLR4-KO astrocytes treated with or without ethanol (40 mM) for 24 h. Flow cytometry, nanoparticle tracking analysis technology, combined with exosomal molecular markers (tetraspanins) along with electron microscopy, were used to characterize and quantify EVs. The content of EVs in inflammatory proteins, mRNA, and miRNAs was analyzed by Western blot and RT-PCR in both astrocyte-derived EVs and the neurons incubated or not with these EVs. Functional analyses of miRNAs were also performed.

**Results:**

We show that ethanol increases the number of secreted nanovesicles and their content by raising the levels of both inflammatory-related proteins (TLR4, NFκB-p65, IL-1R, caspase-1, NLRP3) and by changing miRNAs (mir-146a, mir-182, and mir-200b) in the EVs from the WT-astrocytes compared with those from the untreated WT cells. No changes were observed in either the number of isolated EVs or their content between the untreated and ethanol-treated TLR4-KO astrocytes. We also show that astrocyte-derived EVs could be internalized by naïve cortical neurons to increase the neuronal levels of inflammatory protein (COX-2) and miRNAs (e.g., mir-146a) and to compromise their survival. The functional analysis of miRNAs revealed the regulatory role of the expressed miRNAs in some genes involved in several inflammatory pathways.

**Conclusions:**

These results suggest that astrocyte-derived EVs could act as cellular transmitters of inflammation signaling by spreading and amplifying the neuroinflammatory response induced by ethanol through TLR4 activation.

**Electronic supplementary material:**

The online version of this article (10.1186/s12974-019-1529-x) contains supplementary material, which is available to authorized users.

## Background

Astrocytes, one of the most abundant cells in the central nervous system, have emerged as important regulators of brain function as they participate under physiological and pathological conditions. Indeed, astrocytes support the neuronal and synaptic function by providing trophic support, and by regulating the extracellular environment, cerebral blood flow, neurotransmitter synthesis, free radical formation, etc. However, dysfunction of astrocytes is also involved in the pathophysiology of neurological disorders, including neurodegenerative disease, stroke, epilepsy, migraine, and neuroinflammatory diseases [1].

The nature of astrocyte-to-neuron communication is mediated by direct cell-to-cell contact, and also by the astrocyte secretome that differs according to physiological or pathological conditions [2]. One important astrocyte secretome component is exosomes, which are membrane-derived microvesicles secreted by most cell types [3–5]. Originally, when exosomes or extracellular vesicles (EVs) were identified for the first time, they were assigned the function of removing unnecessary proteins from the cell [6]. However, recent evidence supports the role of exosomes in different physiological processes, such as intercellular communication, proliferation, and immune response, and under neuropathological conditions, such as neurodegenerative diseases like Alzheimer’s, Parkinson’s, or prion diseases [7–9].

The functional implications of these EVs depend on the milieu of proteins, mRNA, miRNA, tRNA, and lipids that they carry in their interior or are embedded in their membrane [10]. Indeed, the proteomic, lipidomic, and RNA profiles inside EVs are extensively affected upon cell stress or pathological insults when their functional outcome varies on target cells [11]. Similarly, EVs also contain miRNAs or small non coding RNA molecules, which are involved in the post-transcriptional regulation of gene expression [12]. The relevance of the miRNAs present inside EVs is crucial as they can markedly alter the transcriptome of target cells and change their physiological state [13].

Immune surveillance in the central nervous system is achieved mainly through the interplay between microglia and astroglia. These cells express an array of innate immune receptors to detect and respond to pathogens, tissue, or cellular damage, and other factors [14]. Among immune receptors, toll-like receptor (TLR), a type of transmembrane protein located either in the plasma or the endosome membrane, constitutes an important family of receptors with 10 or 12 members (in humans or mice, respectively) [15]. One interesting member of this family is toll-like receptor 4 (TLR4), whose classical agonist is lipopolysaccharide (LPS), a major Gram-negative bacterial cell wall component [16]. TLR4 activation triggers a signaling cascade that promotes nuclear factor kappa-light-chain-enhancer of activated B cells (NFκB) nuclear translocation, where it acts as a transcription factor of several genes involved in inflammation. The transcription of these genes leads to the secretion of proinflammatory cytokines, chemokines, and microvesicles [17–20].

However, TLR4 can also recognize some endogenous compounds and responds to cellular damage or damage-associated molecular patterns (DAMPs) [21]. We have also demonstrated that alcohol, by interacting with *lipid rafts*, is also capable of triggering the TLR4 signaling response in glial cells [22, 23] by, in turn, triggering neuroinflammation and neural damage in animals after acute or chronic alcohol exposure [14, 24, 25]. The genetic or pharmacological blockade of the TLR4 response prevents the neuroinflammatory damage caused by ethanol in both glial cells and animal models [14, 24, 25]. However, the mechanisms of the maintenance and transmission of the ethanol-induced inflammatory response in the brain remain elusive.

By considering the relevance of EVs in brain communication, the present study explores the potential role of astroglial EVs as intercellular mediators of the ethanol-induced inflammatory response and the role of TLR4 in these events. Here, we report that astrocytes treated with ethanol are capable of increasing both the secretion of EVs and the content of inflammatory-related proteins (e.g., TLR4, cytokines) by changing the levels of miRNAs compared with untreated astrocytes. Strikingly, ethanol-induced changes were abolished in the EVs from ethanol-treated TLR4-deficient astrocytes. We provide further evidence that the EVs primed from ethanol-treated WT astrocytes are able to target and alter the physiological state of neurons by internalizing their inflammatory content, events that may contribute to spread neuroinflammation.

## Methods

### Animals

C57/BL6 wild-type (WT), TLR4-Knock-out (TLR4-KO) (C57/BL6 background, kindly provided by Dr. S. Akira, Osaka, Japan), and transgenic β actin DsRed mice (ACTB-DsRed) (The Jackson Laboratory, MI, USA) were used. Mice were distributed into 3–4 animals per cage, separated by genotypes. They were maintained with water and solid diet ad libitum under controlled conditions of temperature (23 °C), humidity (60%), and light/dark cycles (12 h/12 h). After mating, pregnant females were placed inside separate cages during the gestation period. Then fetuses (17-day-old) and newborn mice were sacrificed by decapitation to perform the neuronal culture and the astroglial culture, respectively. All the experimental procedures were carried out in accordance with the guidelines approved by the European Communities Council Directive (86/609/ECC) and by Spanish Royal Decree 1201/2005 with the approval of the Ethical Committee of Animal Experimentation of the Príncipe Felipe Research Centre (Valencia, Spain).

### Primary culture of astrocytes, ethanol treatment, and the isolation of astrocyte-derived EVs

Astroglial cells (98 ± 0.5% GFAP-positive cells) [26] were obtained from brain cortices of new born WT and TLR4-KO pups (6–8 animal per culture). They were mechanically dissociated and cultured in Dulbecco’s modified eagle’s medium (DMEM, Lonza, Belgium), supplemented with 20% fetal bovine serum (FBS), 100 U/mL penicillin/streptomycin, 2.5 μg/mL fungizone, 2 mM glutamine, and 1 g/L glucose, and seeded at 850 cells/mm^2^. On day 7 in vitro, FBS was reduced to 10% and glucose was removed. On day 14 in vitro, cell cultures were 90–95% confluent and FBS was replaced with bovine serum albumin (BSA, 1 mg/mL) 24 h prior to ethanol (40 mM) stimulation to avoid EVs from being present in FBS. After 24 h of ethanol stimulation, both media and cells were harvested.

For the isolation of astrocyte-derived EVs, media were spun at 17,000×*g* and 4 °C for 10 min. Supernatants were collected, filtered using 0.22 μm needle filters (Acrodisc Syringe Filter, Pall, UK), and transferred to fresh tubes before being spun at 100,000×*g* for 2 h at 4 °C. The pellets containing an exosome-rich fraction were resuspended in PBS-containing protease inhibitors. Protein levels were determined by the BCA assay and the amount of mg protein/mL under the different experimental conditions was adjusted to ~ 10 mg/mL.

### Exosome characterization by transmission electron microscopy

The freshly isolated EVs derived from astrocyte culture media were fixed with 2% para-formaldehyde and were prepared as previously described [27]. Preparations were examined under a transmission FEI Tecnai G2 Spirit electron microscope (FEI Europe, Eindhoven, The Netherlands) using a digital camera Morada (Olympus Soft Image Solutions GmbH, Münster, Germany).

### Nanoparticles tracking analysis

An analysis of the absolute size range and concentration of microvesicles was performed using NanoSight NS300 Malvern (NanoSight Ltd., Minton Park, UK). Particles were automatically tracked and sized-based on Brownian motion and the diffusion coefficient. After isolation, astrocyte-derived EVs were re-suspended in 0.8 mL of 0.22 μm-filtered PBS. The measurement conditions of the nanoparticles tracking analysis were temperature = 25 ± 0.5 °C, viscosity = 0.99 ± 0.01 cP, frames per second = 25, and measurement time = 30 s. The detection threshold was similar for all the samples. Additional file 2: Figure S2B provides an example of the graph obtained in each experiment using size distribution vs. microvesicles concentration.

### Western blot analysis of EVs

For the Western blot analysis, equal volumes of astrocyte-derived EVs were separated in SDS-PAGE gels and transferred to PVDF membranes as previously described [23, 28]. The used antibodies were anti-CD63, anti-CD9, anti-CD81, anti-TLR4, anti-NLRP3, anti-IL-1R, anti-NFκB-p65 (nuclear transcription factor-κB), anti-caspase-1, and anti-calnexin (Santa Cruz Biotechnology, USA). Membranes were incubated with the respective anti-HRP secondary antibodies and developed with the ECL system (ECL Plus; Thermo Scientific, Illinois, USA). Band intensity was quantified by the ImageJ 1.44p analysis software, and the densitometric analysis is shown in arbitrary units normalized to CD63 (Fig. 2) or GAPDH (Fig. 6) as loading controls.

### Flow cytometry analysis

The freshly isolated astrocyte-derived EVs were diluted in the 0.22 μm-filtered PBS and were then stained under sterile dark conditions with green-RNA-binding, a liposoluble fluorophore SYTO (Syto RNA Select Green, Invitrogen, USA) that is able to cross the EV membrane. Samples were vortexed and bathed at 37 °C in the dark for 30 min before being loaded into the flow cytometer CytoFlex S (Beckman and Coulter, USA) and visualized by the CytExpert software. The cytometer was washed with detergent and water between the EVs samples to eliminate any remaining residue between samples. CytoFLEX was set up to detect microvesicles using Megamix-Plus FSC beads (BioCytex, ref. 7820), this being a mixture of 100 nm, 300 nm, 500 nm, and 900 nm fluorescent beads. The FITC fluorescence of these beads was analyzed using the 488 nm laser and 525 nm fluorescence emission filter. Moreover, the forward scatter (FSC) and side scatter (SSC) signals from 488 nm laser, the 405 nm side scatter (violet side scatter, VSSC), were used to improve the detection of small particles. In the dot plot FITC/VSSC, fluorescent beads were visualized by adjusting the gains of the fluorescence and scatter detectors. These gains were applied to acquire the samples stained with BODIPY (see Additional file 2: Figure S2).

### RNA isolation, reverse transcription, and quantitative RT-PCR

The total RNA of EVs was isolated and cleansed following the manufacturer’s instructions (Total Exosome RNA Isolation Kit, Invitrogen, Lithuania; RNeasy MinElute Cleanup, Qiagen, Germany). A bioanalyzer was used to quantify RNA purity and concentration (Agilent Technologies, Santa Clara, CA, USA) (Additional file 3: Figure S3). Total mRNA and total miRNA were reverse-transcribed using the Transcriptor First Strand cDNA synthesis kit (Roche Diagnostics, Mannheim, Germany) and TaqMan Advanced miRNA assays (ThermoFisher Scientific, USA), respectively.

Quantitative two-step RT-PCR (real-time reverse transcription) was performed in the Light-Cycler 480 detection System (Roche Diagnostics). Genes were amplified using the SYBR Green PCR Master Mix (Roche Diagnostics) following the manufacturer’s instructions (Roche Diagnostics). The mRNA level of housekeeping gene cyclophilin A was used as an internal control for normalization. Specific miRNAs were amplified by the TaqMan Fast Advanced Master Mix (ThermoFisher Scientific). Data were analyzed using the LightCycler 480 relative quantification software. RNAU6 was used as an internal control for the normalization of the miRNAs levels in neurons. The nucleotide sequences of the primers used to detect the presence of several miRNA and RNA are shown in Additional file 4: Table S1 and Table S2.

### Internalization of astroglial EVs by cortical neurons in culture

The neurons (purity of neurons ~ 95%) [26] derived from the brain cortices of the 16-day-old WT and ACTB-DsRed transgenic embryos were seeded in poly-d-lysine-tissue coated culture wells and maintained with Neurobasal medium supplemented with B-27 (Invitrogen, USA) under controlled conditions of temperature, humidity, and CO_2_ for 3–4 days, as described elsewhere [23].

For the internalization studies, neurons were plated on 12-mm glass coverslips. On day 3 in vitro*,* they were incubated overnight with 10 μL of the fresh astrocyte-derived EVs previously stained with red-fluorophore lipid-binding Bodipy (Bodipy TR Ceramide, Invitrogen, USA) if added to the naïve WT neurons, or with green-RNA-binding fluorophore SYTO (Syto RNA Select Green, Invitrogen, USA) if added to the ACTB-DsRed neurons. The double staining for either EVs or neurons was performed to minimize the technical flaws of each fluorophore. The naïve WT neurons were stained with Cell Tracker, 5 μM (Invitrogen, Oregon, USA), for 30 min. These neurons, along with the ACTB-DsRed neurons, were fixed with 3.7% paraformaldehyde in PBS (with Ca^2+^ and Mg^2+^) for 20 min, and permeabilized with 0.1% NP-40 for 5 min. Nuclei were stained with 0.5 μg/mL Hoechst 33342 dye (Molecular Probes). Coverslips were mounted in FA mounting fluid (Difco, Madrid, Spain). Fluorescence images were quantified in single cells under a Leica confocal microscope (model TCS-SP8-AOBS, Mannheim, Germany). The fluorescence intensity of EVs was measured by LAS AF Lite (Leica Confocal Software, USA), and the results were expressed as the fluorescence intensity per cell (arbitrary units). Some neurons from the ACTB-DsRed transgenic mice were grown on 25-mm glass coverslips (Menzel-Gläser, Braunschweig, Germany). Figure 5b corroborates that EVs were internalized in the cortical neurons using the xyz axes projection.

The primary cultures of the naïve mice cortical neurons were also incubated with 10 μL of the fresh astrocyte-derived EVs for 24 and 48 h for the mRNA analysis and the protein analysis, respectively. At the end of the incubation period, neurons were washed several times with PBS, and were collected to be analyzed by Western blot and quantitative RT-PCR (mRNA and miRNA). The protein levels of the fresh astrocyte-derived EVs and the neuronal lysate were determined by the BCA assay. The apoptotic nuclei staining with Hoechst 33342 dye was evaluated and the nuclear fragmentation in 500–750 cells per experimental condition was quantified.

### Bioinformatic analysis of miRNAs

The miRNA functional enrichment analysis was performed using different webserver tools. DIANA miRPath, v2.0 was employed (*diana.imis.athenainnovation*.*gr*/*DianaTools*), a bioinformatic tool that allows different miRNAs to be overlaid to identify the most significant KEGG pathways (Kyoto Encyclopedia of the Genes and Genomes) related with the target genes. *mirnet.ca* (http://diana.imis.athena-innovation.gr/) webserver [29, 30] was also used, an application which generates targets that derive from microarray, RNAseq, or RT-qPCR experiments, and allows those genes potentially regulated by the analyzed miRNAs to be identified. The bioinformatic webserver STRING (*http*://*www*.*string*-*db*.*org*) was employed to provide interactions across matched proteins by generating protein-protein interaction networks [31].

## Results

### The ethanol-triggered secretion of EVs by astrocytes is dependent on the TLR4 response

**Fig. 1:**
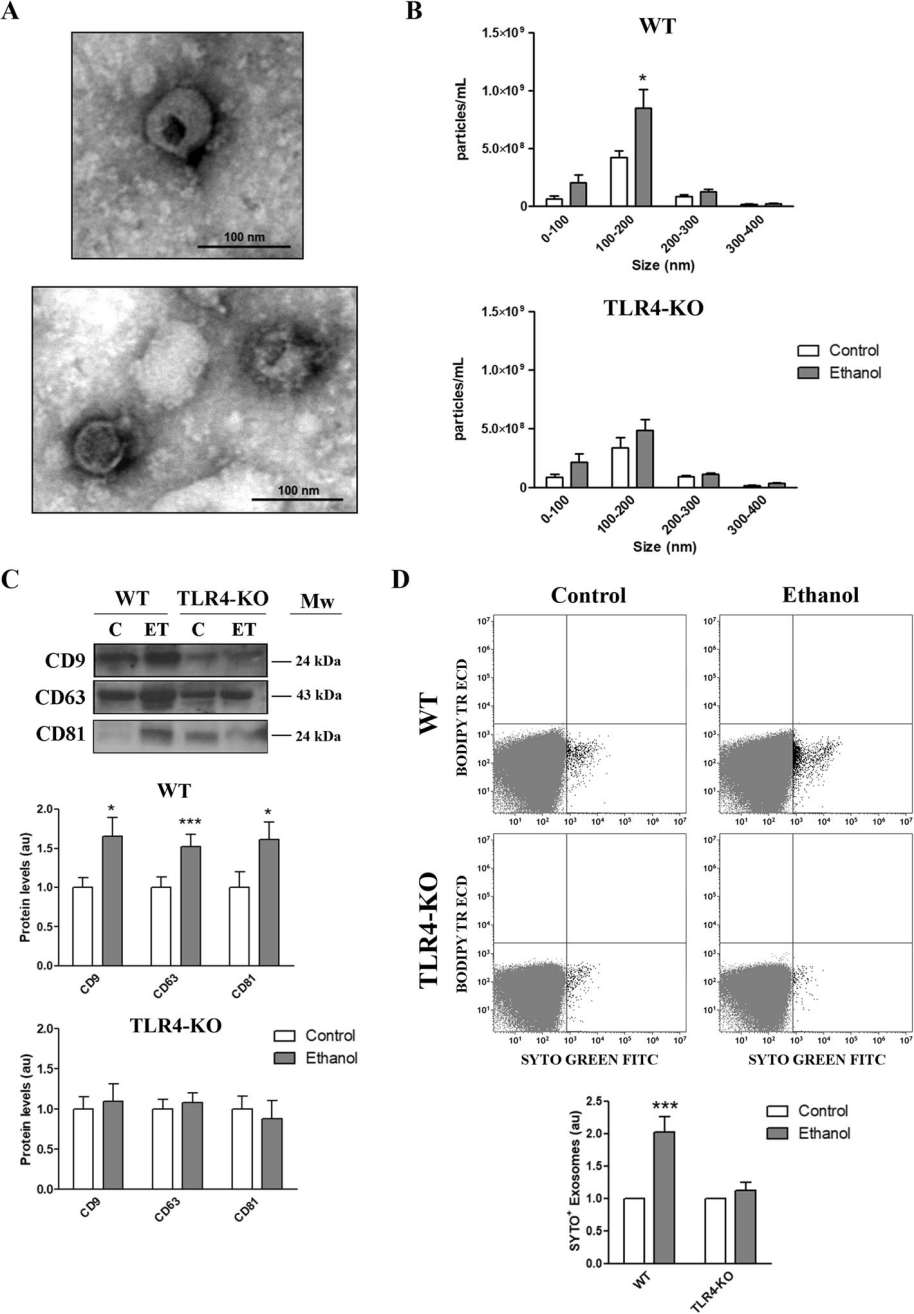
Characterization of the EVs secreted by the untreated and ethanol-treated WT and TLR4-KO astrocytes. **a** Electron microscopy images of astrocyte-derived EVs. Scale bar, 100 nm. **b** Measurement of the absolute size range and concentration of the microvesicles derived from astrocytes by the nanoparticles tracking analysis. **c** Immunoblot analysis and quantification of CD9, CD63, and CD81 in astrocyte-derived EVs. A representative immunoblot for each protein and their molecular weight are shown. 40 μg/μL of protein were loaded in each lane. **d** Flow cytometry analysis and quantification of the astrocyte-derived EVs stained with green-RNA-binding fluorophore SYTO (SYTO^+^ events are black-colored). The WT and TLR4-KO astroglial cells were treated with ethanol (40 mM) for 24 h. Data represent mean ± SEM, *n* = 5–7 independent experiments. *p < 0.05 and ***p < 0.001 compared to their respective control group, according to the two-way ANOVA followed by Bonferroni’s post-hoc test

To evaluate whether ethanol is able to increase the secretion of EVs from astroglial cells, the cultured cortical astrocytes from the WT or TLR4-KO mice were treated with or without ethanol (40 mM) for 24 h. Then the EVs released in the culture medium by astrocytes were isolated by ultracentrifugation. The transmission electron microscopy study revealed that the particles obtained from both groups displayed typical exosome characteristics in size and shape terms. However, as we were uncertain about all the nanoparticles being exosomes (around 100 nm in diameter), we decided to refer to them as EVs (Fig. 1a). We further analyzed the size distribution and concentration of the astrocyte-secreted nanoparticles using NanoSight. The results showed that the highest peak of the secreted nanoparticles ranged between 100 and 200 nm, which includes the size range of EVs (Fig. 1b). These structures are highly enriched in different proteins named tetraspanins, which can be used as a marker of exosome secretion. Figure 1c shows that ethanol is able to increase the secretion of EVs in the WT astrocytes as the same number of astrocytes triggers higher levels of EV markers, such as tetraspanins CD9, CD63, and CD81 in the ethanol-treated WT astrocyte-derived EVs compared to the levels of these proteins in the EVs of the untreated astrocytes. Notably, the absence of calnexin in astrocyte-derived EVs confirmed the absence of cytosolic protein contamination (Additional file 1: Figure S1). Likewise, flow cytometry and the SYTO green fluorescent nucleic acid stain, which labels the RNA contained in EVs, confirmed that ethanol increased the number of the SYTO-positive nanoparticles in astrocyte-derived EVs (Fig. 1d).

We also used EVs isolated from the ethanol-treated and untreated TLR4-KO astrocytes. Notably, no changes were observed in the levels of tetraspanins (Fig. 1c), the number of the SYTO-positive nanoparticles measured by cytometry (Fig. 1d), or in the NanoSight analyzer results (Fig. 1b) compared to the EVs derived from the untreated and ethanol-treated TLR4-KO astrocytes, which suggests the role of the TLR4 response in these events.

### Ethanol treatment alters the content of proteins and miRNAs associated with inflammation in the EVs that derived from the WT astrocytes, but not in the EVs derived from the TLR4-KO astrocytes

**Fig. 2:**
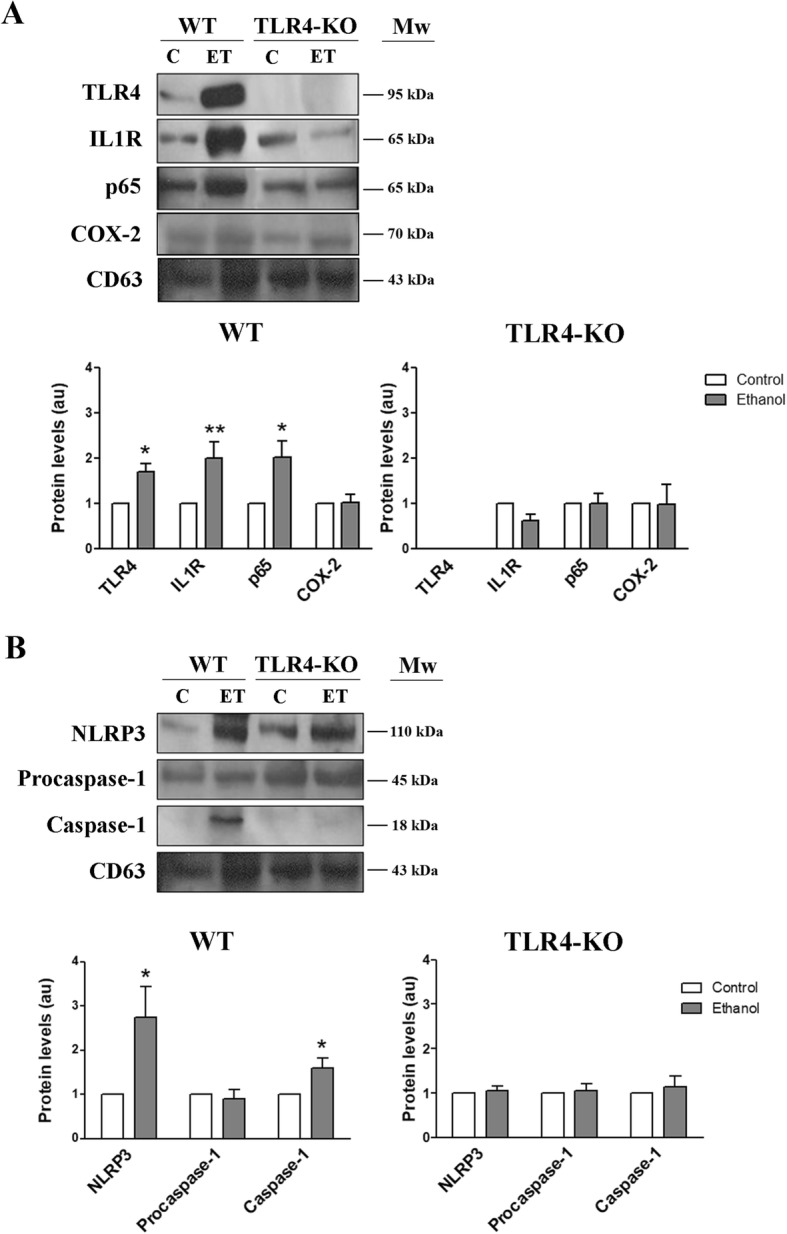
Effects of ethanol on the levels of the different proteins in the WT and TLR4-KO astrocyte-derived EVs. Immunoblot analysis and quantification of the following proteins: **a** TLR4, IL-1R, COX-2, NFκB-p65; **b** NLRP3, procaspase-1, and caspase-1 of the EVs secreted by the untreated and ethanol-treated WT and TLR4-KO astrocytes. CD63 was used as a loading control. The WT and TLR4-KO astroglial cells were treated with ethanol (40 mM) for 24 h. A representative immunoblot for each protein and their molecular weight are shown. 40 μg/μL of protein were loaded in each lane. Data represent mean ± SEM, *n* = 6 independent experiments. *p < 0.05 and **p < 0.01 compared to their respective control group, according to the two-way ANOVA followed by Bonferroni’s post-hoc test. For the TLR4 statistical analysis, *p < 0.05 compared with the control value, according to the *t* test

Our previous studies demonstrated that ethanol triggers an inflammatory response via the activation of the TLR4 signaling pathway [22, 32] and NOD-like receptor NLRP3-inflammasome [33] in astroglial cells. Therefore, we evaluated whether ethanol treatment could alter the content of astrocyte-derived EVs by affecting the enrichment/levels of the proteins and miRNAs involved in neuroinflammation. Figure 2a, b shows that ethanol treatment increased the protein content of TLR4, IL-1R, and the NFκB-p65 subunit, as well as the NLRP3 and active caspase-1, in astrocyte-derived EVs compared to the control counterparts (Fig. 2a, b). These proteins were also present in the lysate of astrocytes in culture, as previously reported [22, 32, 33]. Conversely, no changes were observed in the protein content and levels of IL-1R, NFκB-p65, NLRP3, and active caspase-1 between the ethanol-treated and untreated TLR4-KO astrocyte-derived EVs (Fig. 2a, b). As expected, TLR4 was not detected in the EVs from the TLR4-KO astrocytes.

**Fig. 3:**
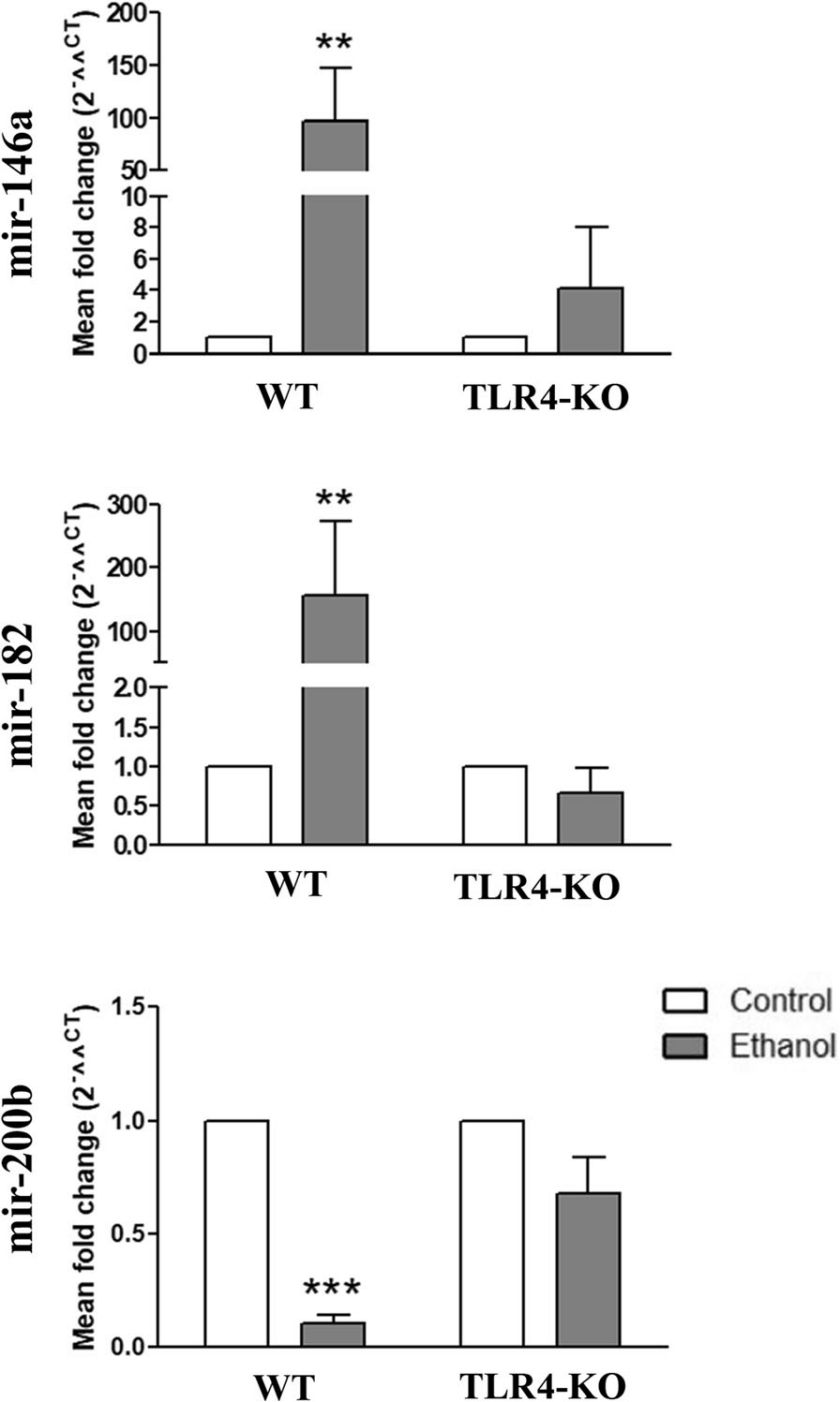
Effects of ethanol on the differential levels of miRNAs in the EVs derived from the untreated and ethanol-treated WT and TLR4-KO astrocytes. The WT and TLR4-KO astroglial cells were treated with ethanol (40 mM) for 24 h. Data represent mean ± SEM, *n* = 3–6 independent experiments. **p < 0.01 and ***p < 0.001 compared to their respective control counterparts, according to the two-way ANOVA followed by Bonferroni’s post-hoc test

It is well established that miRNAs, noncoding small RNAs, play a key role in the regulation of gene expression [34], and are involved in many pathologies, including neuroinflammatory conditions and degenerative diseases [35]. Indeed, we recently showed that in vivo ethanol treatment alters the expression of specific miRNAs associated with neuroinflammation in mice cerebral cortices [36]. Therefore, we evaluated some of the miRNAs affected by the in vivo ethanol treatment in astrocyte-derived EVs, such as mir-146a [12], mir-182 [37], and mir-200b [38]. By a qRT-PCR analysis, we showed that in vitro ethanol treatment significantly increased the levels of mir-182 and mir-146a levels, while mir-200b lowered in the EVs from the ethanol-treated WT astrocytes (Fig. 3). However, no changes were found in the EVs derived from the ethanol-treated and untreated TLR4-deficient astrocytes. These results suggest that some miRNAs involved in inflammation could modulate the immune receptors and signaling molecules in astrocyte-derived EVs and may extend neuroinflammation.

### Bioinformatic analysis of miRNAs in astrocyte-derived EVs

**Fig. 4:**
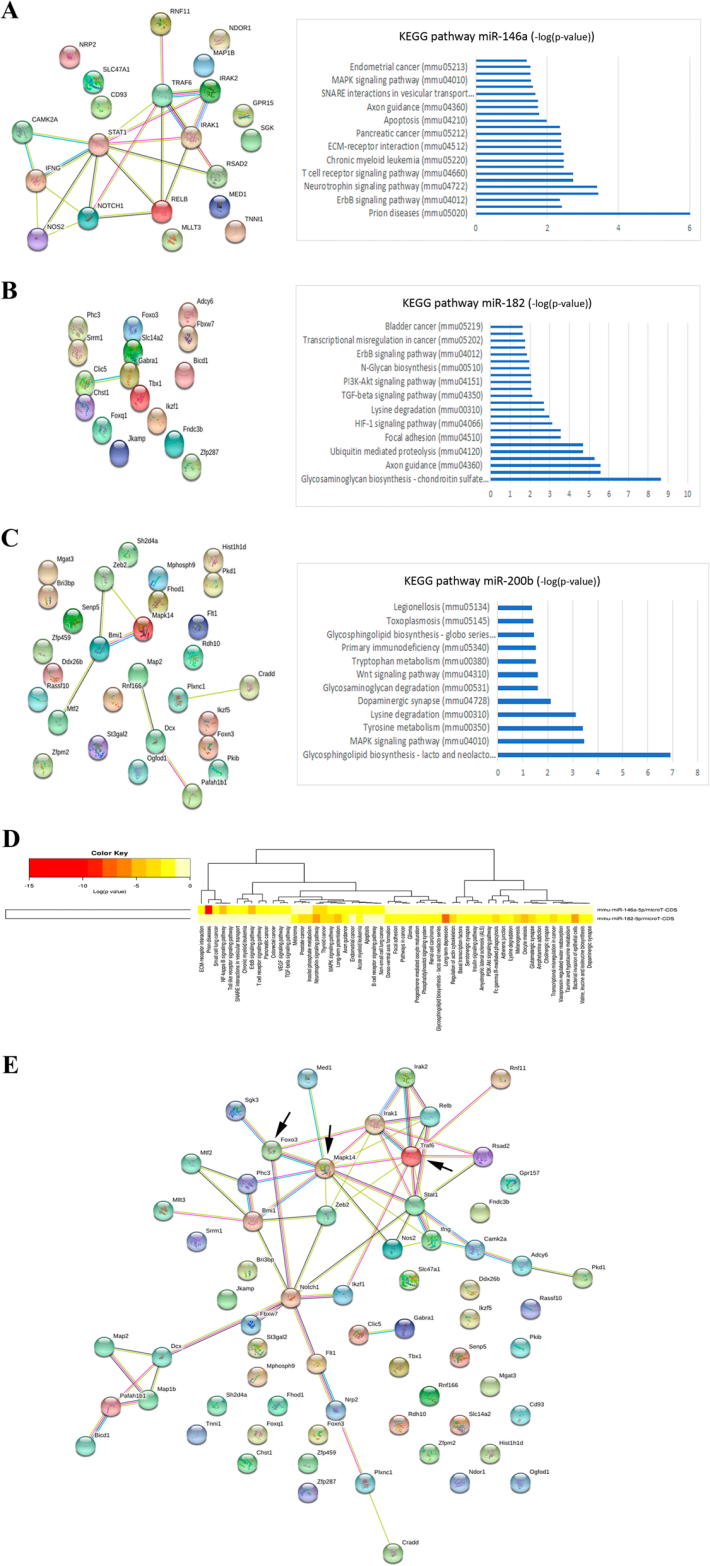
Functional analysis of mmu-mir-146a, mmu-mir-182, and mmu-mir-200b. **a**–**c** The protein-protein interaction for the predicted genes of each miRNA and the principal KEGG pathways modulated by the STRING protein proteins network interaction analysis performed with the STRING webserver. The *p* values are represented as (-log(*p* value)). **d** Heatmap of the modified principal KEGG pathways derived from the genes simultaneously affected by mir-146a and mir-182. **e** The protein-protein interaction for the predicted genes simultaneously modulated by mir-146a and mir-182 by the STRING webserver database. Arrows indicate the genes selected for the gene expression analysis shown in Fig. 7b

After confirming that ethanol treatment changed the levels of some miRNAs, we performed a functional analysis for these noncoding RNAs (mir-146a, mir-182, and mir-200b) to further establish the main biological functions and key pathways involved in alcohol effects and the TLR4 immune response. The bioinformatic analysis was separated into different steps. The first step determined the preferential targets modulated by each miRNA using the (www.mirnet.ca) webserver (Additional file 4: Table S3). Then with the identified targets, the STRING (http://www.string-db.org) webtool was used to assess the protein-protein interaction network. Figure 4a–c reveals how mir-146a and mir-200b displayed a stronger interaction of the modulated targets than mir-182. This figure also shows the significantly affected pathways according to their *p* value and in relation to these targets. All the affected pathways are included in Additional file 4: Table S4.

By using the *DIANA miRPath v2.0 tool*, which allows the metabolic signaling pathways that are cooperatively affected by miR-146a and miR-182 to be identified, we also recognized several signaling pathways, such as NFκB, TGF-β, MAP-Kinases, axonal growth, among others (see heatmap Fig. 4d and Additional file 4: Table S5), which are modulated by miRNAs. Finally, from the targets jointly modulated by mir-146a and mir-182, we performed a protein-protein interaction analysis using the *String* database. Figure 4d shows that the TLR4-related genes (proteins), such as Irak1, Traf6, and Mapk14, formed strong interaction points between different proteins. These results indicated that mir-146a and mir-182 simultaneously modulated inflammatory signaling pathways.

### Role of the TLR4 response in the induction of inflammatory proteins and miRNAs in the cortical neurons incubated with the ethanol-treated astrocyte-derived EVs

**Fig. 5:**
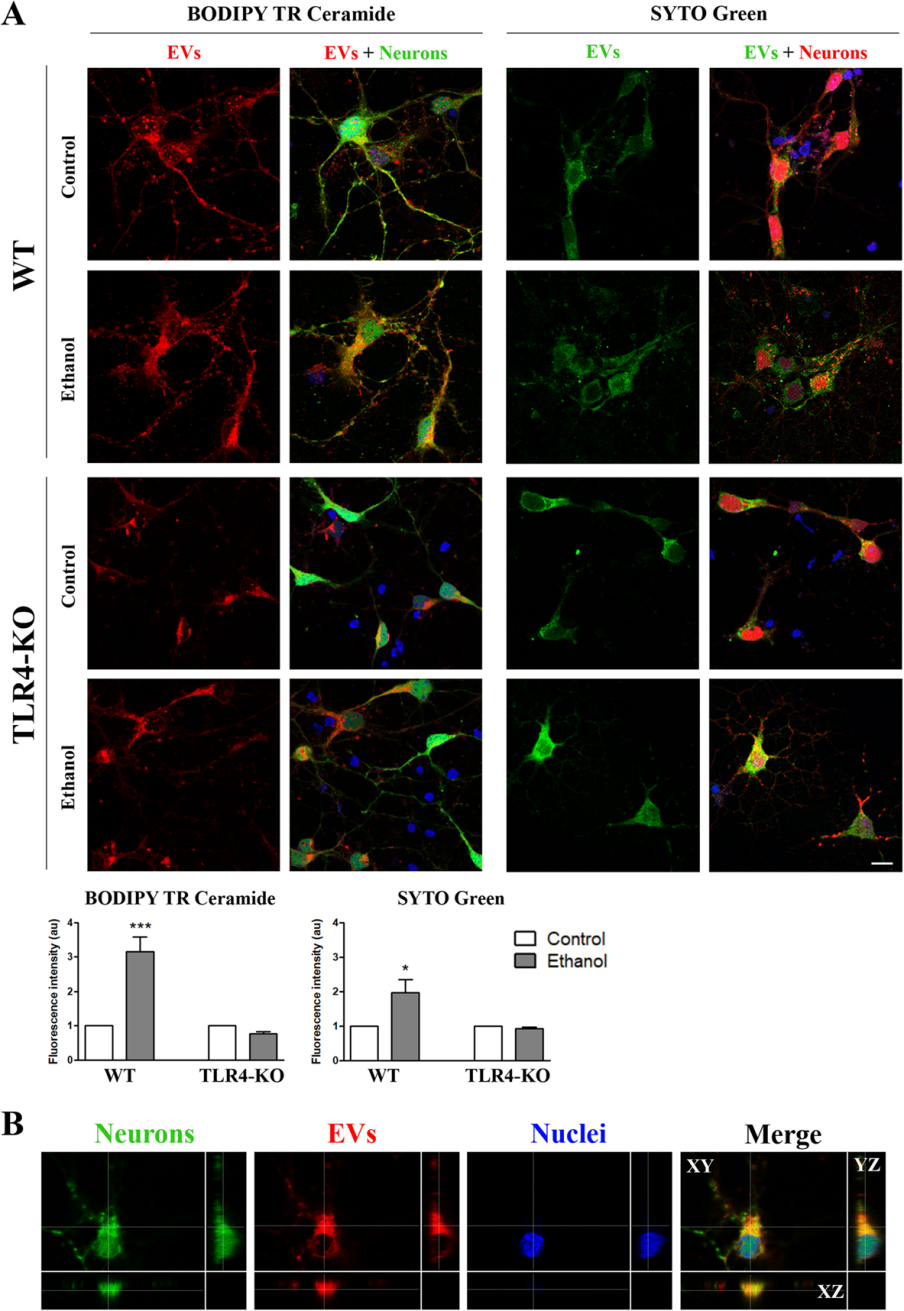
Analysis of the internalization of the astrocyte-derived EVs in naïve cortical neurons. Neurons were incubated overnight with 9–10 μg/μL of the fresh astrocyte-derived EVs. **a** Fluorescence intensity of the red-fluorophore lipid-binding Bodipy or the green-RNA-binding fluorophore SYTO astrocyte-derived EVs internalized by green-stained (Cell Tracker) or red (ACTB-Ds Red) naïve cortical neurons, respectively. A representative photomicrograph of each condition is shown. Scale bar, 10 μm. EVs were obtained from the WT and TLR4-KO astroglial cells treated with ethanol (40 mM) for 24 h. Bar graphs represent the mean ± SEM of the data from three different fields per condition from six distinct cultures. **b** Confocal microscopy images showing that EVs are internalized in the neuronal cytoplasm, as demonstrated using the xyz axes projections. Green, red, and blue fluorescence represent the cell tracker, the EVs stained with Bodipy, and nuclei staining, respectively. *p < 0.05 and ***p < 0.001 compared to their respective control group, according to the two-way ANOVA followed by Bonferroni’s post-hoc test

As EVs play an important role in transmitting cell-to-cell information during homeostasis and under pathological conditions [39], we assessed the potential transfer and internalization of cortical astrocyte-derived EVs to cortical neurons in primary culture. For these experiments, we used neurons from either DS-Red transgenic or naïve mice, which were incubated with the EVs pre-stained with either SYTO or Bodipy, respectively. Figure 5b clearly shows that EVs are internalized by neurons, as demonstrated for their accumulation (Bodipy staining) in the neuronal cytoplasm. We also observed greater fluorescence intensity (SYTO or Bodipy) in the neurons incubated for 24 h with the EVs derived from the ethanol-treated astrocytes (Fig. 5a) compared to the control counterparts. Interestingly, when these cells were incubated with the EVs that derived from the TLR4-KO astrocytes, we found no changes between the ethanol-treated and untreated groups in either Bodipy or SYTO staining (Fig. 5a).

**Fig. 6:**
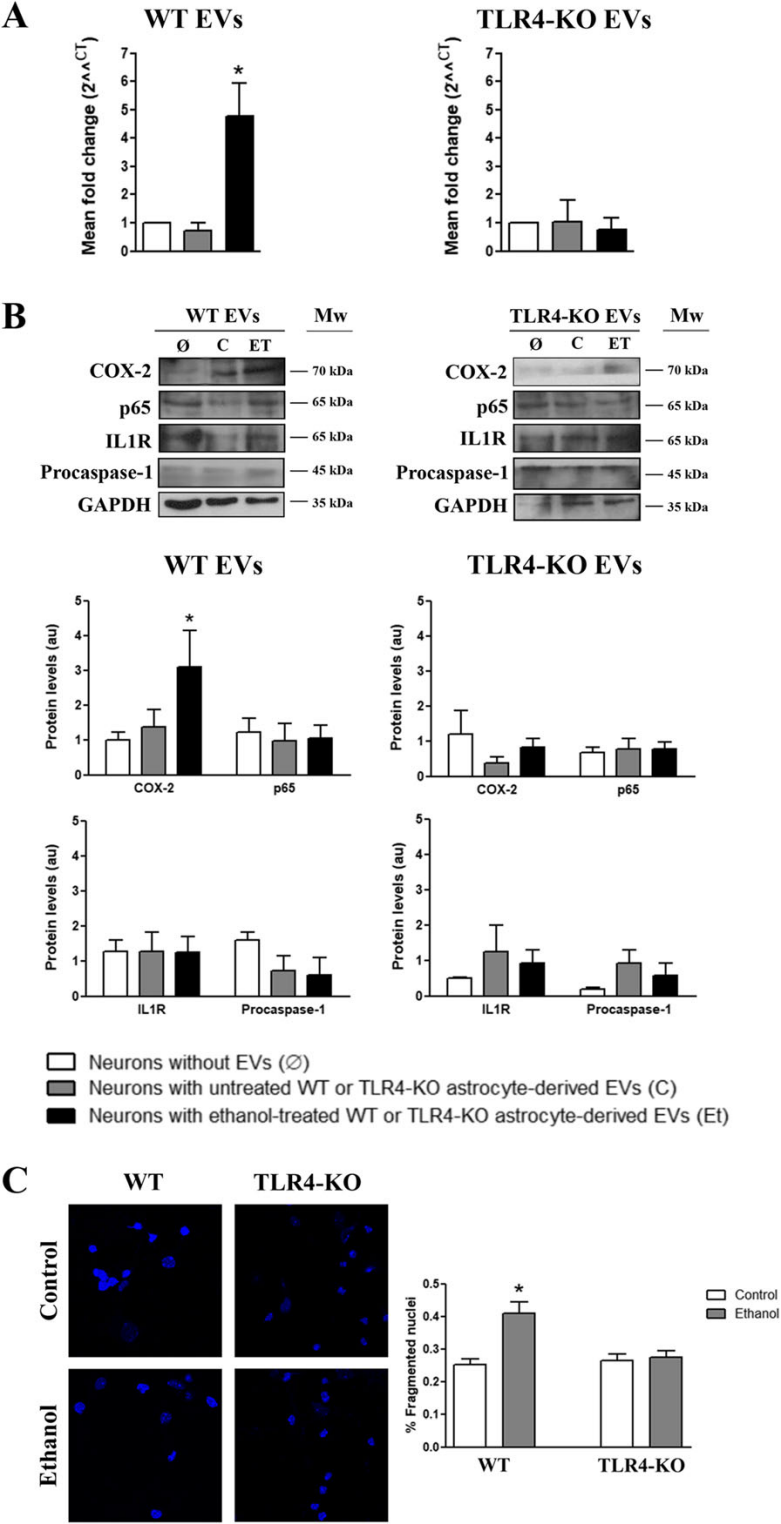
Effects of the cortical astrocyte-derived EVs internalization on primary cortical neurons. Neurons were incubated overnight with 9–10 μg/μL of the fresh astrocyte-derived EVs. **a** Analysis of the neuronal mRNA expression of IL-1β in the presence of the EVs derived from the untreated and ethanol-treated (40 mM, 24 h) WT and TLR4-KO astrocytes. **b** Immunoblot analysis and quantification of the neuronal levels of COX-2, NFκB-p65, IL-1RI, and procaspase-1 in the presence of the EVs derived from the untreated or ethanol-treated (40 mM, 48 h) WT and TLR4-KO astrocytes. A group of untreated neurons with EVs was also added to the experiments. A representative immunoblot for each protein and their molecular weight are shown. 40 μg/μL of protein were loaded in each lane. GAPDH was used as a control loading. **c** Confocal microscopy analysis of the fragmented nuclei of neurons stained with Hoechst 33342 dye. 500–750 nuclei per condition were quantified. Bar graphs represent the percentage of fragmented nuclei of neurons per genotype and treatment. Data represent mean ± SEM, *n* = 4–5 independent experiments. *p < 0.05 compared to their respective control counterparts according to the one-way ANOVA followed by Bonferroni’s post-hoc test

We next investigated whether EVs internalization could alter the content/levels of inflammatory proteins and genes in neurons. We observed that the neuronal uptake of the ethanol-treated WT astrocyte-derived EVs significantly increased the protein expression of COX-2 and the mRNA levels of IL-1β after a 48-h and a 24-h incubation period, respectively (Fig. 6a, b). No significant changes were observed in the levels of other proteins (NFκB-p65, IL-1RI and procaspase-1). Strikingly, the internalization of astrocyte-derived EVs treated with ethanol significantly enhanced apoptotic neuronal death (Fig. 6c), as demonstrated by the increasing percentage of fragmented nuclei (41.0 ± 3.6%) compared to the neurons incubated with the EVs from astrocytes not treated with ethanol (25.4 ± 1.7%). Figure 6a, b also shows that no changes in the aforementioned protein and mRNA expression took place in the cortical neurons incubated with the EVs from the ethanol-treated and untreated TLR4-KO astrocytes. Likewise, the internalization of the TLR4-KO astrocyte-derived EVs did not induce neuronal apoptosis in either the neurons incubated with EVs derived from ethanol-treated (27.4 ± 2.2%) or the untreated (26.5 ± 1.9%) TLR4-KO astrocytes (Fig. 6c).

**Fig. 7:**
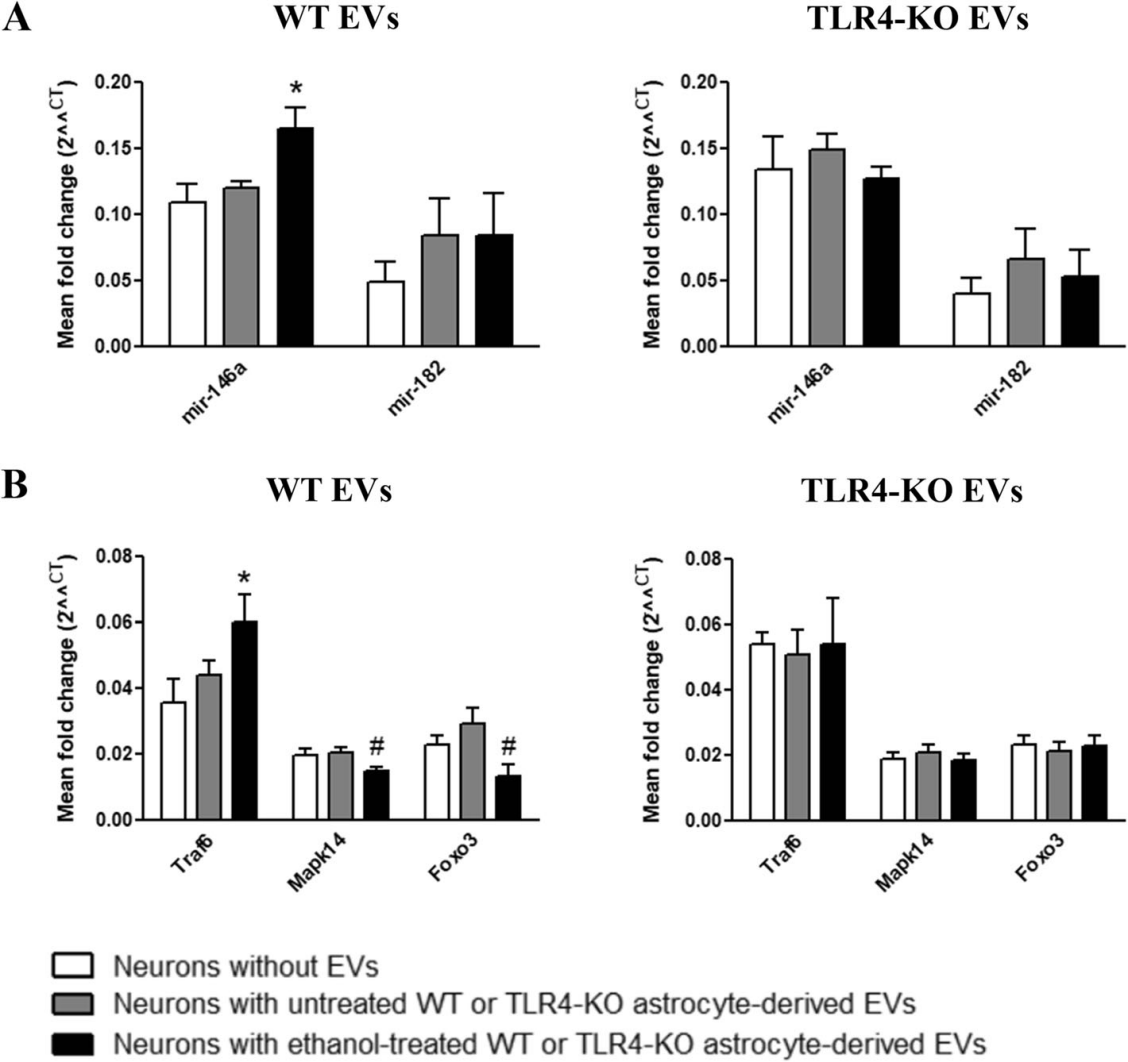
The ethanol-treated, WT astrocyte-derived EVs increased the mir-146a levels in the primary cultures of neurons. **a** Results of the expression levels for mir-146a and miR-182 by TaqMan advance RT-qPCR in the cultures of the neurons incubated with the EVs that derived from the WT and TLR4-KO astrocytes treated or not with ethanol. A group of untreated neurons with EVs was also added to the experiments. The results are expressed in arbitrary units, with normalizing compared to RNAU6. Data represent mean ± SEM, *n* = 4–5 independent experiments. *p < 0.05 compared to the neurons without EVs according to the one-way ANOVA followed by Bonferroni’s post-hoc test. **b** Gene expression analysis by RT-qPCR of some genes selected from the miRNA functional analysis, Traf6, Mapk14, and Foxo3. The results are expressed in arbitrary units, with normalizing versus Cyclophilin A. Data represent mean ± SEM, *n* = 4–5 independent experiments. *p < 0.05 compared to the neurons without EVs, #*p* < 0.05 compared to their respective control counterparts according to the *t* test

We then wondered whether the changes observed in the proteins and genes expression (Fig. 6) in the neurons incubated with astrocyte-derived EVs could be associated with the changes in the levels of miRNAs. Notably, a significant increase in the mir-146a levels took place in the neurons incubated with the ethanol-treated WT astrocyte-derived EVs compared to the neurons incubated or not with the control WT astrocyte-derived EVs. However, mir-182 displayed a slight, but not significant, increase in the neurons incubated with either the ethanol-treated or untreated WT astrocyte-derived EVs (Fig. 7a). The expression of mir-200b in the cultured neurons and neuronal cells incubated with EVs was very low, so we did not conduct further analyses (data not shown).

We next assessed whether the transfer of miRNAs from astrocyte-derived EVs to neurons could affect some target genes in the neurons incubated with the ethanol-treated and untreated astrocyte-derived EVs. For this purpose, we analyzed the specific target genes of miRNAs. The functional analysis of miRNAs predicted the potential targets associated with miRNAs overexpression (Fig. 4d). With this analysis, we identified that the expression of Traf6, Mapk14, and Foxo3 (Fig. 7b) was modified in the cultures of the neurons incubated with the EVs derived from the ethanol-stimulated astrocytes. However, these changes were not observed in the neurons incubated with the EVs from either the control WT astrocytes or the TLR4-KO astrocytes. It should be noted that this analysis also showed other predicted targets, such as Notch1, TNF-α, and Irak1, whose mRNA levels did not change (data not shown).

Finally, if we consider that not all the proteins or miRNAs enriched as cargoes of EVs altered in neuronal extracts, the results suggest that this is a response of neurons to internalized EVs rather than a direct measure of EVs’ enrichment profile.

## Discussion

Current evidence demonstrates the relevance of exosomes or EVs in intercellular communication by them participating in both physiological and pathological events [40, 41], and as mediators of neuroinflammation associated with several neuropathologies [42]. We have shown that binge ethanol drinking in adolescence causes neuroinflammation and brain damage by a mechanism involved in the TLR4 response. However, the role that glial EVs play in ethanol-induced neuroinflammation and their potential relation with TLR4 signaling remain unexplored. We herein demonstrate for the first time that ethanol treatment alters the secretion and content of the EVs from the WT astroglial cells by increasing the cargo of the protein associated with the TLR4 and NLRP3 pathways, as well as inflammatory-related miRNA. We also show that astrocyte-derived EVs can be internalized by cortical neurons, which affects the physiological state of recipient cells by altering the levels of some inflammatory-related proteins (COX-2), genes (IL-1B, Traf6, Mapk14, and Foxo3), and miRNA (mir-146a), which can trigger apoptosis. These results support the notion that astroglia-derived EVs play a role in spreading neuroinflammation. The results also support the role of the TLR4 response because no ethanol effects were observed in the EVs that derived from the TLR4-KO astrocytes.

Different studies have demonstrated that by following different stimuli, cells are capable of increasing their EVs secretion. For instance, more EVs and changes in their content have been observed in macrophages after LPS stimulation [43, 44]. Ethanol also enhances the number of EVs secreted by primary human monocytes and THP-1 monocytic cells in concentration- and time-dependent manners [45]. Using cortical astrocytes in culture, we demonstrate that ethanol not only increases the number of secreted EVs but also alters their content and enrichment in proinflammatory proteins, such as TLR4, IL-1R, NFκB-p65, NLRP3, and caspase-1. These proteins are important regulators of innate immune defense, recognized pathogen-associated molecular patterns (PAMPs) and DAMPs. Thus TLR4 and IL-1R initiate the immune response through the activation of NFκB by triggering the induction of cytokines and chemokines, while NLRP3 and active caspase-1 are core components of the inflammasome, which facilitates the processing of pro-IL-1β to the active form of IL-1β [46]. These results support our previous findings, which showed that ethanol can activate TLR4, IL-1R [14], and the NLRP3 signaling response [33] in culture astrocytes. In line with our results, a recent study demonstrated that ethanol can trigger the release of the microvesicles containing the let-7b/HMGB1 complexes that derive from BV2 microglial cells [47].

The relevance of astrocytes-neuron communication through the release of EVs, and their involvement as carriers of protective or neurotoxic molecules, has been demonstrated in recent years. For instance, astrocyte-derived EVs containing Synapsin I [48] or prion protein [10] protect neurons after ischemia or hypoxia, which leads to improved neuronal survival and neurite outgrowth. Conversely, astrocyte-derived EVs can also transport misfolded pathogenic proteins and/or aberrantly expressed miRNAs, which act as initiators and propagators of neuroinflammation [42] and neural death [49]. Several studies have also shown a close relationship between apoptosis processes and the release of the EVs containing specific miRNAs involved in cell death [50, 51]. The present findings demonstrated that the EVs from the ethanol-treated astrocytes carry proinflammatory proteins (e.g., NFκB-p65, NLRP3, caspase-1, IL-1β) and miRNAs, and that these elements may trigger neuronal apoptotic cell death. These results suggest that glial EVs might initiate and amplify the neuronal inflammatory response by leading to neuronal dysfunction and brain damage.

EVs are also enriched in miRNAs and can modify the cellular phenotype and/or physiology of recipient cells [52]. Although few effects of ethanol on EVs miRNA content in neural cells have been described, clinical studies have demonstrated that ethanol treatment upregulates mir-122 in the EVs from human hepatocytes and liver mononuclear cells [53]. The upregulation of mir-192 and mir-30a has also been found in the plasma of alcoholic hepatitis patients [54]. We have recently shown that chronic ethanol treatment triggers the dysregulation of cluster 182 (mir182-mir183-mir96) and mir-200a/b expression in mice cerebral cortices [36]. These miRNAs regulate the innate immune response as mir-200b/c is involved in the TLR4/NFκB response [38], while mir-146a and mir-182 participate in innate immunity and inflammation [37, 55]. In the present study, we show that astrocyte-derived EVs present differential levels of mir-182, mir-146a, and mir-200b miRNAs. Our bioinformatics analysis indicated that mir-146a and mir-182 performed joint action with some of the targets involved in the signaling molecules associated with the TLR4 response, including Irak1, Traf6, Mapk14, and Map-kinases [56, 57]. We also provide evidence that the miRNAs transferred from astrocyte-derived EVs to neurons can affect some target genes modulated by mir-146a and mir-182, such as Traf6 and Mapk14, which were significantly affected in the neurons incubated with the ethanol-treated astrocyte-derived EVs. These results suggest that ethanol can promote changes in the miRNA content of astroglia EVs, and their transfer to neurons could increase inflammatory-related genes and proteins, which might cause neuronal damage and death, as demonstrated in the neurons incubated with the ethanol-treated WT astrocyte-derived EVs.

In summary, we provide evidence for the role of glial EVs in extending neuroinflammation and neuronal dysfunctions in alcohol neuropathology and addiction.

## Conclusions

Our results reveal for the first time that ethanol increases the release of astrocyte-derived EVs and their content enriched in inflammation-related proteins and miRNAs, and that this mechanism is dependent on the TLR4 signaling response. We also identify that, upon ethanol treatment, astrocytes are able to propagate an inflammatory response to cortical neurons through EVs, events that might contribute to neuronal dysfunction and neuroinflammation. This study opens up new avenues to understand the potential role of EVs to initiate or amplify the neuroinflammatory response induced by ethanol.

## Additional files

Additional file 1: **Figure S1.** Immunoblot analysis of the calnexin levels present in the EVs from the untreated and ethanol-treated WT and TLR4-KO astrocytes. The absence of the calnexin expression in the exosome samples confirmed the absence of cytosolic protein contamination. A sample of astrocyte lysate was used as positive control of the calnexin expression. (TIF 489 kb)

Additional file 2: **Figure S2.** A) Flow cytometry graph of a mixture of FITC fluorescent beads with different diameters of 100 nm, 300 nm, 500 nm and 900 nm (Megamix-Plus FSC beads), which was used to detect the EVs obtained from the WT and TLR4-KO astrocytes. B) Example of the graph obtained in the nanoparticles tracking analysis using size distribution and the concentration of microvesicles. (TIF 924 kb)

Additional file 3: **Figure S3.** Analysis of the RNA population isolated from the WT and TLR4-KO, ethanol-treated or not astrocyte-derived EVs by a 2100 Agilent Bioanalyzer. X axis shows the nucleotide length of the RNA population and the Y axis its fluorescence intensity. (TIF 366 kb)

Additional file 4: **Table S1.** Nucleotide sequences of the primers used for the TaqMan RT-qPCR of miRNAs. **Table S2.** Nucleotide sequences of the primers used for the RT-PCR of genes. **Table S3.** Targets for mmu-mir-146a, mmu-mir-182 and mmu-mir-200b obtained by the mirnet.es webserver. **Table S4.** The KEGG pathways obtained by the DIANA tool webserver. **Table S5.** The KEGG pathways that derived from the *String* protein-protrin interaction analysis between the target genes modulated by mmu-miR-146a and mmu-mir-182. (DOCX 57 kb)

## Abbreviations

ACTB-DsRed: Transgenic β actin DsRed mice
DAMSs: Damage-associated molecular patterns
EVs: Extracellular vesicles
LPS: Lipopolysaccharide
NFκB: Nuclear factor kappa-light-chain-enhancer of activated B cells
PAMS: Pathogen-associated molecular patterns
PND: Postnatal day
qRT-PCR: Quantitative RT-PCR
TLR4: Toll-like receptor 4
TLR4-KO: TLR4-knockout
WT: Wild-type
