# Supplementary figures and images for: TLR4 participates in the transmission of ethanol-induced neuroinflammation via astrocyte-derived extracellular vesicles
Source: J Neuroinflammation. 2019 Jul 4;16:136. doi: 10.1186/s12974-019-1529-x (PMC6610989; doi:10.1186/s12974-019-1529-x)

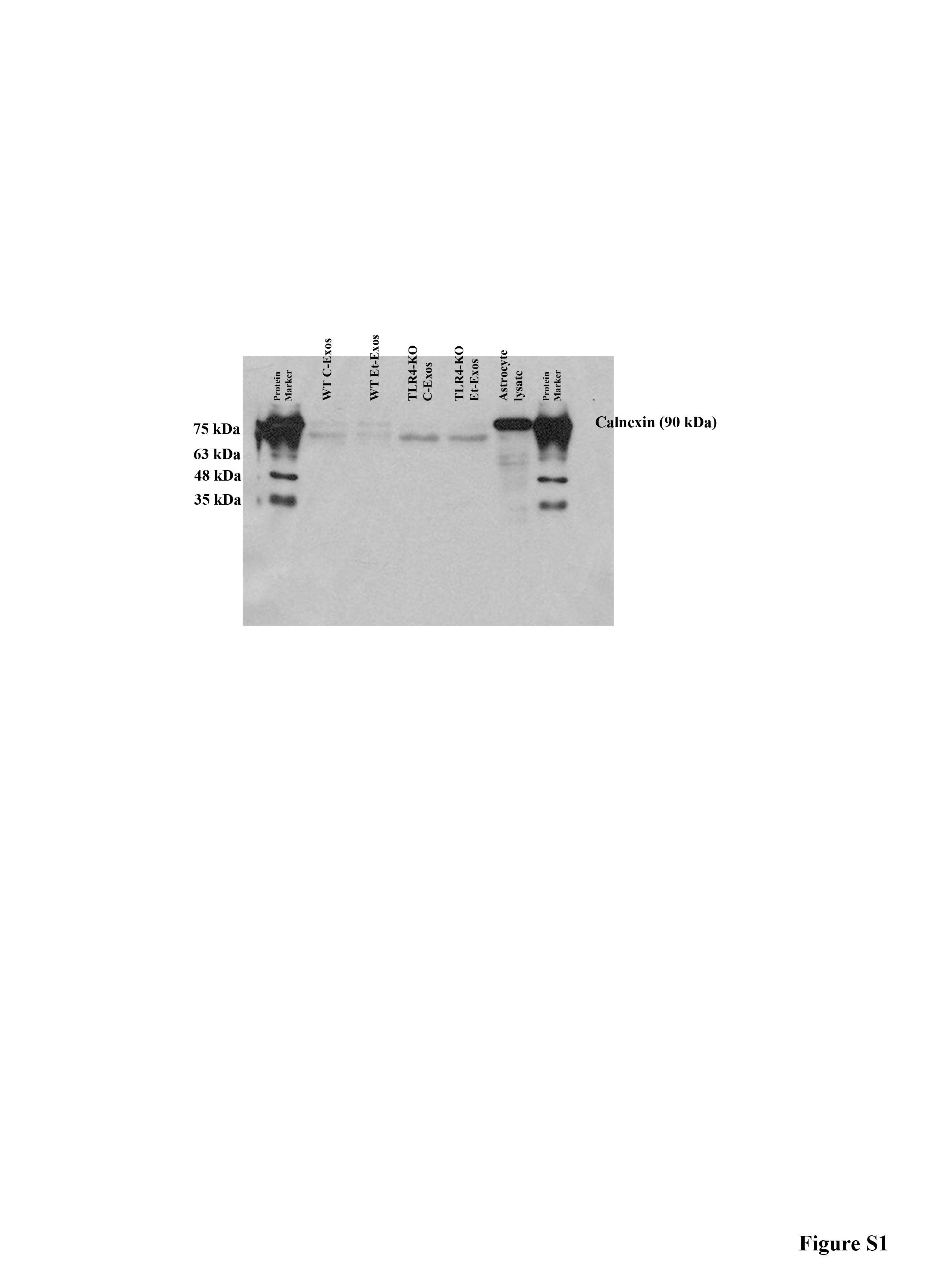

Supplement: Supplementary file 1 — Figure S1. Immunoblot analysis of the calnexin levels present in the EVs from the untreated and ethanol-treated WT and TLR4-KO astrocytes. The absence of the calnexin expression in the exosome samples confirmed the absence of cytosolic protein contamination. A sample of astrocyte lysate was used as positive control of the calnexin expression. (TIF 489 kb) [file 12974_2019_1529_MOESM1_ESM.tif]

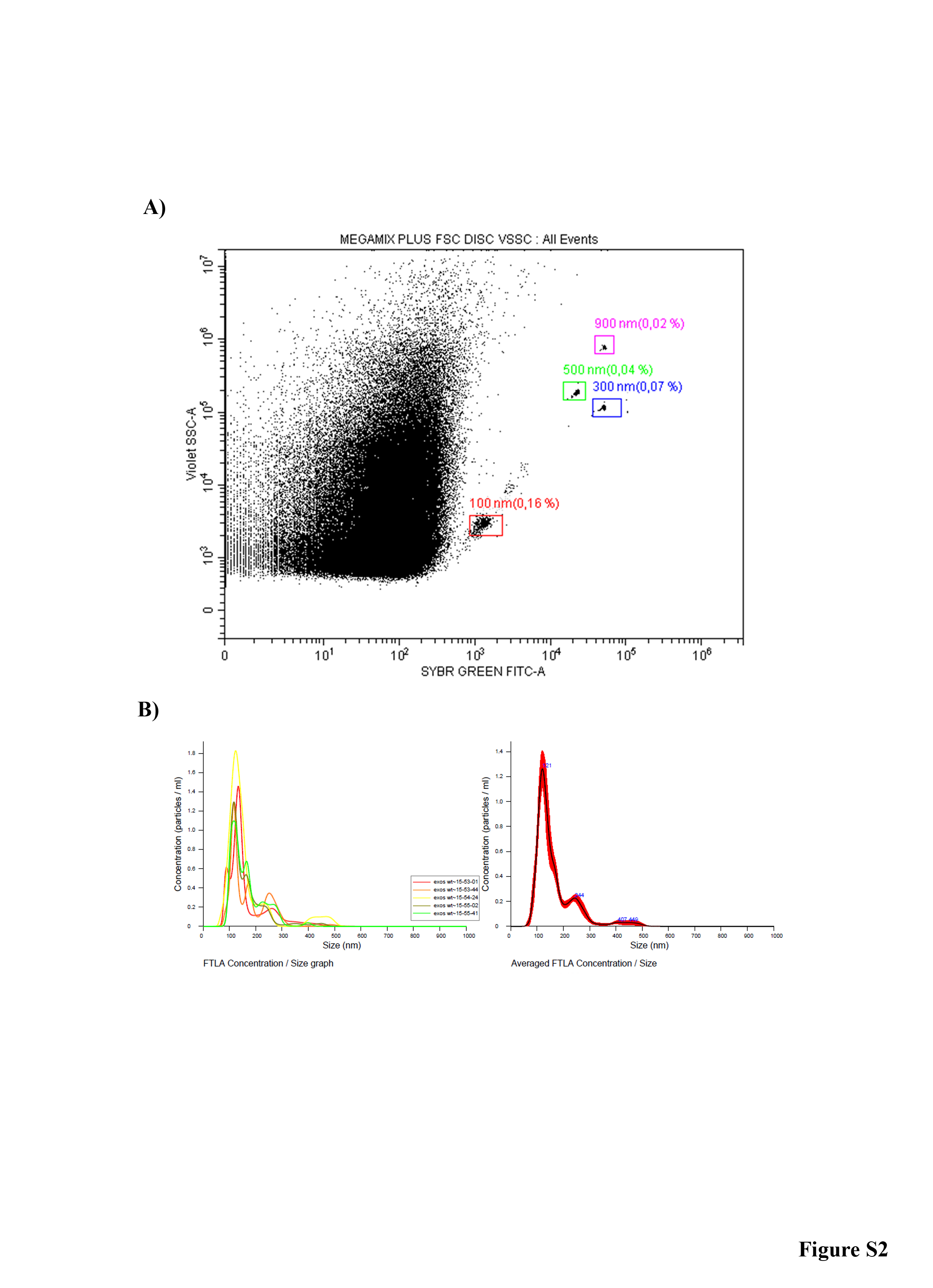

Supplement: Supplementary file 2 — Figure S2. A) Flow cytometry graph of a mixture of FITC fluorescent beads with different diameters of 100 nm, 300 nm, 500 nm and 900 nm (Megamix-Plus FSC beads), which was used to detect the EVs obtained from the WT and TLR4-KO astrocytes. B) Example of the graph obtained in the nanoparticles tracking analysis using size distribution and the concentration of microvesicles. (TIF 924 kb) [file 12974_2019_1529_MOESM2_ESM.tif]

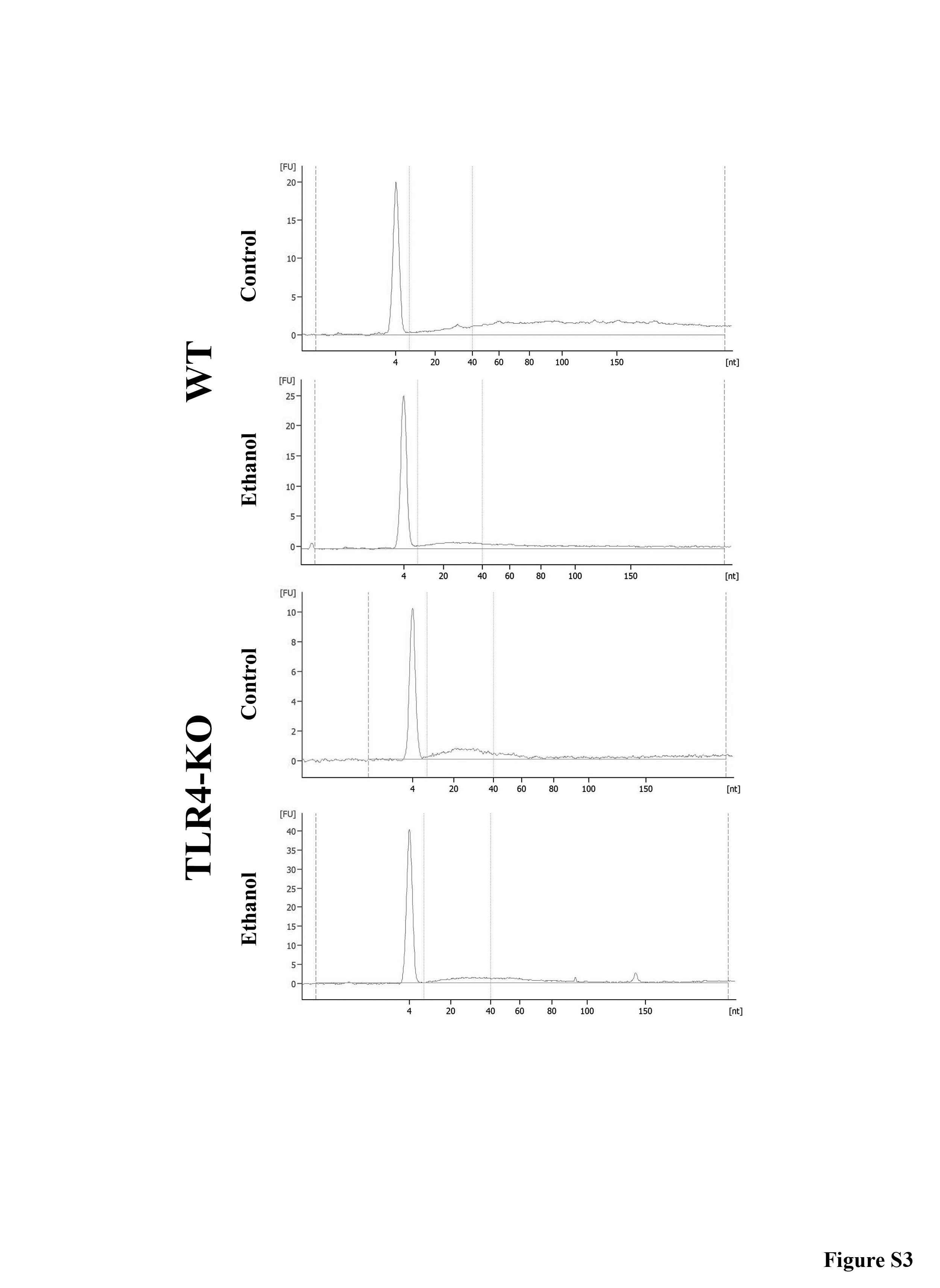

Supplement: Supplementary file 3 — Figure S3. Analysis of the RNA population isolated from the WT and TLR4-KO, ethanol-treated or not astrocyte-derived EVs by a 2100 Agilent Bioanalyzer. X axis shows the nucleotide length of the RNA population and the Y axis its fluorescence intensity. (TIF 366 kb) [file 12974_2019_1529_MOESM3_ESM.tif]
