# Supplementary material for: TLR4 participates in the transmission of ethanol-induced neuroinflammation via astrocyte-derived extracellular vesicles
Source: J Neuroinflammation. 2019 Jul 4;16:136. doi: 10.1186/s12974-019-1529-x (PMC6610989; doi:10.1186/s12974-019-1529-x)
Supplement: Supplementary file 4 — Table S1. Nucleotide sequences of the primers used for the TaqMan RT-qPCR of miRNAs. Table S2. Nucleotide sequences of the primers used for the RT-PCR of genes. Table S3. Targets for mmu-mir-146a, mmu-mir-182 and mmu-mir-200b obtained by the mirnet.es webserver. Table S4. The KEGG pathways obtained by the DIANA tool webserver. Table S5. The KEGG pathways that derived from the String protein-protrin interaction analysis between the target genes modulated by mmu-miR-146a and mmu-mir-182. (DOCX 57 kb) [file 12974_2019_1529_MOESM4_ESM.docx]

**Additional file 4**

**Table S1:** Nucleotide sequences of the primers used for TaqMan RT-qPCR of miRNAs.

| miRNA | miRBase Accession Number/ NCBI Accession Number* | Primer sequences (5’ to 3’) |
| --- | --- | --- |
| mmu-miR-146a | MIMAT0000158 | UGAGAACUGAAUUCCAUGGGUU |
| mmu-mir-182 | MIMAT0000211 | UUUGGCAAUGGUAGAACUCACACCG |
| mmu-mir-200b | MIMAT0004545 | CAUCUUACUGGGCAGCAUUGGA |
| U6 snRNA | NR_004394* | GTGCTCGCTTCGGCAGCACATATACTAAAATTGGAACGATACAGAGAAGATTAGCATGGCCCCTGCGCAAGGATGACACGCAAATTCGTGAAGCGTTCCATATTTT |

**Table S2:** Nucleotide sequences of the primers used for RT-PCR of genes.

| Gene | Primer sequences (5’ to 3’) |
| --- | --- |
| IL-1β | F: ACAGAATATCAACCAACAAGTGATATTCTC  R: GATTCTTTCCTTTGAGGCCCA |
| Cyclophilin A | F: GTCTCCTTCGAGCTGTTTGC  R: GATGCCAGGACCTGTATGCT |
| Foxo3 | F: ACAAACGGCTCACTTTGTCC  R: CCGTGCCTTCATTCTGAAC |
| Traf6 | F: AACGTCCTTTCCAGAAGTGC  R: GAATGTGCAAGGGATTGGAG |
| Mapk14 | F: GACCGTTTCAGTCCATCATTC  R: AACACATCCAACAGACCAATCA |

**Table S3:** Targets for mmu-mir-146a, mmu-mir-182 and mmu-mir-200b obtained by the mirnet.es webserver.

| Targets mmu-miR-146a | Targets mmu-miR-182 | Targets mmu-miR-200b |
| --- | --- | --- |
| Relb | Foxq1 | Pkib |
| Tnni1 | Ikzf1 | Hist1h1d |
| IRAK1 | Slc14a2 | Cradd |
| RNF11 | Srrm1 | Plxnc1 |
| Ndor1 | Jkamp | Rnf166 |
| Rsad2 | Clic5 | Sh2d4a |
| Sgk3 | Zfp287 | Rassf10 |
| Gpr157 | Phc3 | Ddx26b |
| Camk2a | Adcy6 | Mphosph9 |
| Cd93 | Bicd1 | Fhod1 |
| Notch1 | Fbxw7 | Zfp459 |
| Ifng | Fndc3b | Pkd1 |
| Stat1 | Chst1 | St3gal2 |
| Mllt3 | Foxo3 | Bmi1 |
| Irak1 | Gabra1 | Bri3bp |
| Nos2 | Tbx1 | Foxn3 |
| Irak2 |  | Dcx |
| Nrp2 |  | Flt1 |
| Map1b |  | Mapk14 |
| Med1 |  | Mgat3 |
| Traf6 |  | Ikzf5 |
| Slc47a1 |  | Rdh10 |
|  |  | Mtf2 |
|  |  | Pafah1b1 |
|  |  | Senp5 |
|  |  | Ogfod1 |
|  |  | Zfpm2 |
|  |  | Zeb2 |
|  |  | Map2 |

**Table S4:** KEGG pathways, obtained by the DIANA tool webserver.

| **KEGG pathway mmu-miR-146a** | **p-value** | **-LOG(p-value)** | **Num. of genes** |
| --- | --- | --- | --- |
| Prion diseases (mmu05020) | 1,05949E-39 | 38,97490314 | 1 |
| NF-kappa B signaling pathway (mmu04064) | 0,0038 | 2,420216403 | 4 |
| ErbB signaling pathway (mmu04012) | 0,004302628 | 2,366266201 | 5 |
| Thyroid cancer (mmu05216) | 0,000364423 | 3,438393744 | 2 |
| Neurotrophin signaling pathway (mmu04722) | 0,000393469 | 3,405089258 | 5 |
| Toll-like receptor signaling pathway (mmu04620) | 0,001864658 | 2,729400811 | 3 |
| T cell receptor signaling pathway (mmu04660) | 0,001864658 | 2,729400811 | 5 |
| B cell receptor signaling pathway (mmu04662) | 0,00342841 | 2,464907247 | 4 |
| Chronic myeloid leukemia (mmu05220) | 0,00342841 | 2,464907247 | 4 |
| Acute myeloid leukemia (mmu05221) | 0,00342841 | 2,464907247 | 3 |
| ECM-receptor interaction (mmu04512) | 0,003975528 | 2,400605183 | 2 |
| Small cell lung cancer (mmu05222) | 0,003975528 | 2,400605183 | 4 |
| Pancreatic cancer (mmu05212) | 0,003984607 | 2,399614506 | 4 |
| Non-small cell lung cancer (mmu05223) | 0,004296281 | 2,366907321 | 3 |
| Apoptosis (mmu04210) | 0,01040746 | 1,98265525 | 4 |
| VEGF signaling pathway (mmu04370) | 0,01649957 | 1,782527374 | 3 |
| Axon guidance (mmu04360) | 0,01837902 | 1,73567765 | 5 |
| Long-term potentiation (mmu04720) | 0,01837902 | 1,73567765 | 3 |
| SNARE interactions in vesicular transport (mmu04130) | 0,02107917 | 1,676146494 | 2 |
| Colorectal cancer (mmu05210) | 0,02506985 | 1,600848265 | 3 |
| MAPK signaling pathway (mmu04010) | 0,02821486 | 1,5495221 | 6 |
| TGF-beta signaling pathway (mmu04350) | 0,02938917 | 1,531812679 | 2 |
| Endometrial cancer (mmu05213) | 0,02938917 | 1,531812679 | 2 |
| Hepatitis C (mmu05160) | 0,03829618 | 1,416844544 | 4 |

| **KEGG pathway mmu-miR-182** | **p-value** | **-LOG(p-value)** | **Num, of genes** |
| --- | --- | --- | --- |
| Glycosaminoglycan biosynthesis - chondroitin sulfate (mmu00532) | 2,26E-09 | 8,645979389 | 14 |
| MAPK signaling pathway (mmu04010) | 2,56E-06 | 5,592132738 | 10 |
| Axon guidance (mmu04360) | 2,56E-06 | 5,592132738 | 2 |
| Glycosphingolipid biosynthesis - lacto and neolacto series (mmu00601) | 5,23E-06 | 5,281798103 | 9 |
| Ubiquitin mediated proteolysis (mmu04120) | 2,05E-05 | 4,6883008 | 6 |
| Renal cell carcinoma (mmu05211) | 2,05E-05 | 4,6883008 | 10 |
| Focal adhesion (mmu04510) | 0,000270072 | 3,568520439 | 3 |
| Maturity onset diabetes of the young (mmu04950) | 0,000270072 | 3,568520439 | 7 |
| HIF-1 signaling pathway (mmu04066) | 0,000748126 | 3,126025078 | 5 |
| mTOR signaling pathway (mmu04150) | 0,001079945 | 2,966598362 | 3 |
| Lysine degradation (mmu00310) | 0,001794389 | 2,746083402 | 2 |
| Glycosphingolipid biosynthesis - globo series (mmu00603) | 0,00202721 | 2,69310126 | 5 |
| TGF-beta signaling pathway (mmu04350) | 0,007827888 | 2,106355397 | 9 |
| Endocytosis (mmu04144) | 0,008476379 | 2,071789633 | 12 |
| PI3K-Akt signaling pathway (mmu04151) | 0,008995716 | 2,045964264 | 5 |
| Fc gamma R-mediated phagocytosis (mmu04666) | 0,008995716 | 2,045964264 | 3 |
| N-Glycan biosynthesis (mmu00510) | 0,01087105 | 1,963728507 | 6 |
| Neurotrophin signaling pathway (mmu04722) | 0,01087105 | 1,963728507 | 4 |
| ErbB signaling pathway (mmu04012) | 0,01425814 | 1,845937125 | 7 |
| Protein processing in endoplasmic reticulum (mmu04141) | 0,01718307 | 1,764899241 | 8 |
| Transcriptional misregulation in cancer (mmu05202) | 0,01772631 | 1,75138166 | 7 |
| RNA transport (mmu03013) | 0,02267192 | 1,6445117 | 3 |
| Bladder cancer (mmu05219) | 0,02267192 | 1,6445117 | 3 |

| **KEGG pathway mmu-miR-200b** | **p-value** | **-LOG(p-value)** | **Num. of genes** |
| --- | --- | --- | --- |
| Glycosphingolipid biosynthesis - lacto and neolacto series (mmu00601) | 1,22E-07 | 6,91515465 | 1 |
| MAPK signaling pathway (mmu04010) | 3,50E-04 | 3,456353302 | 7 |
| Tyrosine metabolism (mmu00350) | 4,02E-04 | 3,395269184 | 1 |
| Lysine degradation (mmu00310) | 7,55E-04 | 3,122230829 | 2 |
| Dopaminergic synapse (mmu04728) | 7,38E-03 | 2,131664849 | 3 |
| Glycosaminoglycan degradation (mmu00531) | 2,43E-02 | 1,614363345 | 1 |
| Wnt signaling pathway (mmu04310) | 0,02455402 | 1,609877395 | 4 |
| Tryptophan metabolism (mmu00380) | 0,02943236 | 1,531174913 | 1 |
| Primary immunodeficiency (mmu05340) | 0,0297702 | 1,526218248 | 2 |
| Glycosphingolipid biosynthesis - globo series (mmu00603) | 0,03468553 | 1,459851665 | 1 |
| Toxoplasmosis (mmu05145) | 0,03751189 | 1,425831054 | 3 |
| Legionellosis (mmu05134) | 0,04371437 | 1,359375776 | 2 |

P-value and number of genes involved in the pathway were also showed. p-value threshold 0,05, MicroT threshold 0,08 and Fisher’s Exact analysis method.

**Table S5:** KEGG pathways derived from String protein protein interaction analysis between targets genes modulated by mmu-miR-146a and mmu-mir-182.

| Pathway ID | pathway description | count in gene set | false discovery rate |
| --- | --- | --- | --- |
| 05140 | Leishmaniasis | 5 | 8.54e-07 |
| 05152 | Tuberculosis | 6 | 1.14e-06 |
| 05145 | Toxoplasmosis | 5 | 4.87e-06 |
| 05142 | Chagas disease | 4 | 0.000155 |
| 04380 | Osteoclast differentiation | 4 | 0.000253 |
| 05162 | Measles | 4 | 0.000334 |
| 05169 | Epstein-Barr virus infection | 4 | 0.00122 |
| 05133 | Pertussis | 3 | 0.00134 |
| 04064 | NF-kappa B signaling pathway | 3 | 0.00241 |
| 04620 | Toll-like receptor signaling pathway | 3 | 0.00297 |
| 04722 | Neurotrophin signaling pathway | 3 | 0.00404 |
| 04919 | Thyroid hormone signaling pathway | 3 | 0.00404 |
| 05164 | Influenza A | 3 | 0.0105 |
| 05168 | Herpes simplex infection | 3 | 0.0153 |
| 05321 | Inflammatory bowel disease (IBD) | 2 | 0.0248 |
| 05132 | Salmonella infection | 2 | 0.0418 |
| 04210 | Apoptosis | 2 | 0.0423 |
| 05222 | Small cell lung cancer | 2 | 0.044 |
| 05200 | Pathways in cancer | 3 | 0.0455 |
